# Diesel biodegradation capacities of indigenous bacterial species isolated from diesel contaminated soil

**DOI:** 10.1186/s40201-014-0142-2

**Published:** 2014-12-12

**Authors:** Nandhini Palanisamy, Jayaprakash Ramya, Srilakshman Kumar, NS Vasanthi, Preethy Chandran, Sudheer Khan

**Affiliations:** Department of Biotechnology, Bannari Amman Institute of Technology, Sathyamangalam, Tamil Nadu India; CeNTAB, School of Chemical and Biotechnology, SASTRA University, Thanjavur, 613401 Tamil Nadu India

**Keywords:** *Acinetobacter baumannii*, Diesel oil, Parameter optimization, Biodegradation, GC-MS analysis

## Abstract

Petroleum based products are the major source of energy for industries and daily life. Leaks and accidental spills occur regularly during the exploration, production, refining, transport, and storage of petroleum and petroleum products. In the present study we isolated the bacteria from diesel contaminated soil and screened them for diesel biodegradation capacity. One monoculture isolate identified by 16S rRNA gene sequence analysis to be *Acinetobacter baumannii* was further studied for diesel oil biodegradation. The effects of various culture parameters (pH, temperature, NaCl concentrations, initial hydrocarbon concentration, initial inoculum size, role of chemical surfactant, and role of carbon and nitrogen sources) on biodegradation of diesel oil were evaluated. Optimal diesel oil biodegradation by *A. baumanii* occurred at initial pH 7, 35°C and initial hydrocarbon concentration at 4%. The biodegradation products under optimal cultural conditions were analyzed by GC-MS. The present study suggests that *A. baumannii* can be used for effective degradation of diesel oil from industrial effluents contaminated with diesel oil.

## Introduction

Increasing industrial activities and technological progress have demanded a drastic increase in the use of petroleum hydrocarbons. There is considerable risk of environmental contamination during extraction, transportation, refining, storage, usage and ultimate disposal of these non-aqueous phase liquids composed of a large number of hazardous and toxic constituents [1]. Marine and subsurface environment contamination has been reported due to accidental leakage of ships, cars, trucks, etc., leaks and spills from underground storage tank, pipelines and illegal disposals.

Diesel oil, a complex hydrocarbon pollutant is a mixture of alkanes and aromatic compounds that are reported frequently as soil contaminants [2]. One of the best approaches to restoring contaminated soil is to make use of microorganisms able to degrade those toxic compounds in a bioremediation process [3]. Bioremediation is a cost effective approach for cleaning up petroleum hydrocarbons from contaminated area because it is simple to maintain, applicable over large areas and leads to the complete destruction of the contaminant [4]. Bioremediation of these pollutants are significantly affected by the inherent capabilities of the microorganisms to overcome the bioavailability limitations in multiphase environmental scenarios (oil–water–soil) and environmental factors such as temperature, pH, nutrients and electron acceptor availability [5].

The present study employed bacteria isolated from diesel contaminated soil for the biodegradation of diesel oil. Culture parameters for efficient degradation of diesel oil by a bacterial isolate were optimized.

## Materials and methods

### Materials

Diesel oil used in this study was obtained from local petrol bunk, Tamil Nadu, India and sterilized by filter sterilization. All other chemicals used in the present study were of highest purity in grade.

### Isolation, screening and identification

Bacterial species were isolated from diesel contaminated soil (petrol bunk, lorry shed, bus shed and automobile garage) from Sathyamangalam, Tamil Nadu, India [6,7]. Soil samples were serially diluted, 100 μL of the diluted samples spread on the surface of nutrient agar plates and the plates incubated at 37°C for 24 h. The colonies obtained from the agar plates were further sub-cultured to obtain the pure colonies. The bacterial species were screened based on the ability of the bacterial species to degrade diesel oil. The degradation studies were performed in Mineral Salt Medium (MSM) that contains diesel oil as the sole source of carbon. The MSM consists of NH_4_ NO_3_ – 3 g/L, KH_2_PO_4_ - 0.5 g/L, K_2_HPO_4_.3H_2_O - 0.5 g/L and trace amounts of MgSO_4_.7H_2_O - 0.008 g/L, CuSO_4_.4H_2_O - 0.002 g/L, MnSO_4._H_2_O - 0.002 g/L, FeSO_4._7H_2_O - 0.002 g/L and CaCl_2._2H_2_O - 0.002 g/L. The isolated bacterial species from different sites were cultured in Mineral Salt Medium containing diesel oil and incubated for 3 days at 37°C. The growths of the isolated bacterial species were monitored at regular intervals by measuring the optical density at 600 nm. Based on the growth of bacterial species on diesel oil degradation, the best degrader of diesel oil was selected and identified by 16S rRNA gene sequence analysis and used for further studies. Polymerase chain reaction (PCR) was conducted using 16S rRNA forward primer: AGA GTT TGA TCC TGG CTC AG and 16S rRNA reverse primer: ACG GCT ACC TTG TTA CGA CTT. The sequence of the PCR amplicon was submitted to GenBank to obtain accession number. NCBI BLASTN 2.2.26+ comparison software was used to reveal the identities of the isolate.

### Effect of pH

The bacterial species were cultured in Luria Bertani (LB) broth and incubated at 37°C for 24 h. Then the culture was centrifuged at 5000 × g for 10 min, the pellets were collected and washed twice with saline to remove the trace amount of LB medium. To study the effect of pH on degradation, MSM supplemented with 1% of diesel oil was adjusted to different initial pH 4-11 using HCl or NaOH. Two hundred and fifty milliliter Erlenmeyer conical flasks containing 100 mL MSM were inoculated with 2 mL of 1.5 OD inoculum size of strain and incubated at 37°C under shaking condition (120 rpm). In the Control, without the inoculation of bacterial species was kept under similar condition. Samples were collected at regular intervals of time (6 h) up to 120 h.

### Effect of temperature

The bacterial species was cultured in LB broth and was incubated at 37°C for 24 h. Then the culture was centrifuged at 5000 × g for 10 min, the pellets were collected and washed twice with saline to remove the trace amount of LB medium. To study the effect of temperature on degradation, MSM supplemented with 1% of diesel oil was incubated to different temperature such as 25, 30, 35, 40 and 45°C for optimizing the temperature. Two hundred and fifty milliliter Erlenmeyer conical flasks were inoculated with 1.5 optical density inoculum size which was prepared by diluting the bacterial culture with 0.9% NaCl and optical density value was adjusted to 1.5 using colorimeter. Flasks were kept under shaking condition (120 rpm). At regular intervals, the growth of the bacterial species was measured by recording the absorbance at 600 nm. In the control experiments, the flask containing inoculum devoid of diesel oil and the flasks containing diesel oil devoid of inoculum were run parallel at similar conditions.

### Effect of initial hydrocarbon concentration

To study the effect of initial hydrocarbon concentration on degradation, MSM supplemented with different concentrations of diesel oil such as 1-5% was used as substrate. The flasks were inoculated with strain and incubated at 37°C under shaking condition (120 rpm). In the controls, without inoculation of strain were kept under similar conditions with inoculated flasks. Samples were collected at regular intervals of time (6 h) up to 120 h and analyzed for residual diesel oil.

### Effect of initial inoculum size

To study the effect of initial inoculum concentration on degradation, MSM supplemented with 1% of diesel oil was inoculated with different inoculum quantity such as 0.1, 0.6, 1 and 1.5 OD (Absorbance at 600 nm). Two hundred and fifty milliliter Erlenmeyer conical flasks were incubated at 37°C under shaking condition (120 rpm). In the controls, without inoculation of strain were kept under similar conditions with inoculated flasks. Samples were collected at regular intervals of time (6 h) up to 120 h.

### Role of chemical surfactants

To study the role of chemical surfactants on degradation, MSM supplemented with 4% of diesel oil along with 0.02% (w/v)/ (v/v) of different surfactants such as sodium dodecyl sulfate (SDS) and Tween 80 for enhancing the degradation. Two hundred and fifty milliliter Erlenmeyer conical flasks were inoculated with 1.5 OD inoculum size of strain flasks were inoculated with strain and incubated at 37°C under shaking condition (120 rpm). In the controls, without inoculation of strain, were kept under similar conditions with inoculated flasks. Samples were collected at regular intervals of time (6 h) up to 120 h.

### Role of NaCl concentration

To study the role of NaCl concentration on degradation, MSM supplemented with 4% of diesel oil along with different concentrations of NaCl such as 1 mM, 100 mM, 500 mM, 1 M, 2 M and 5 M for checking the salinity condition. Two hundred and fifty milliliter Erlenmeyer conical flasks were inoculated with 1.5 OD inoculum size of bacterial culture. Flasks were inoculated with the bacterial strain and incubated at 37°C under shaking condition (120 rpm). In the controls, without inoculation of strain, were kept under similar conditions with inoculated flasks. Samples were collected at regular intervals of time (6 h) up to 120 h.

### Effect of carbon and nitrogen sources

To study the effect of carbon and nitrogen sources on degradation, MSM supplemented with 4% of diesel oil along with 0.1% of carbon source such as Dextrose, Maltose and 0.1% of nitrogen source such Peptone and Yeast extract. Two hundred and fifty milliliter Erlenmeyer conical flasks were inoculated with 1.5 optical density inoculum size of strain was incubated at 37°C under shaking condition (120 rpm). In the controls, without inoculation of strain, were kept under similar condition with inoculated flasks. Samples were collected at regular intervals of time (6 h) up to 120 h.

### Gas Chromatography- Mass Spectrometry (GC-MS) analysis

GC-MS analysis was done for detecting the degradation effect of diesel oil [8]. After the incubation period, 5 mL of the cultures were extracted with two 20 mL volumes of n-hexane as a solvent by using separating funnels to remove cellular material. The residues were transferred to tarred vials and the volume of each extract was adjusted to 100 mL by adding further n-hexane. The vials were kept at 4°C until the gas chromatographic analysis. Uninoculated control was incubated in parallel to monitor abiotic losses of the substrate.

The degradation effect of diesel oil was detected by GC-MS (Thermo GC- Trace Ultra ver: 5.0, Thermo MS DSQ II), which was equipped with a DB 35- MS Capillary Standard Non-polar column (30 m × 0.25 mm × 0.25 μm). 1 microliter of the organic phase was analyzed by GC-MS. The gas chromatograph equipped with a split–split less injector (split ratios of 50:1) was used for the GC-MS analysis. The oven temperature was initially at 40°C and then programmed to 270°C at a rate of 8°C/min where it was held for 5 min. The temperatures of injector, transfer line and ionization source were all 250°C. The electron impact ionization was tuned at 70 eV and Helium was used as carrier gas with an average linear velocity of 1.0 mL/min.

## Results and discussion

Biodegradation capacity can be evaluated by performing a laboratory study or extensive waste characterization are put together with the bioremediation potential which depends on biodegradability of a specific type of hydrocarbon compound. In this section, isolation of pure cultures and effect of various parameters on hydrocarbon degradation, such as pH, temperature, NaCl concentration, initial hydrocarbon concentration, initial inoculums size, different carbon and nitrogen sources, and chemical surfactant were reported. The roles of biosurfactant effect in contaminant solubilization and biodegradation experiments also have been documented.

### Isolation and identification of bacterial species

The isolated bacterial strain was screened based on the ability to utilize diesel oil. The isolate was identified by 16s rRNA analysis and the organism was *Acinetobacter baumannii* (Accession No. JQ975035)*.* It utilizes hydrocarbons as the sole source of carbon and degrades it to maximum extent. Haritash and Kaushik [9] demonstrated that microorganisms are the main degraders of hydrocarbons. There are various abiotic factors which were optimized for maximum degradation of diesel oil.

### Effect of pH

**Figure 1:**
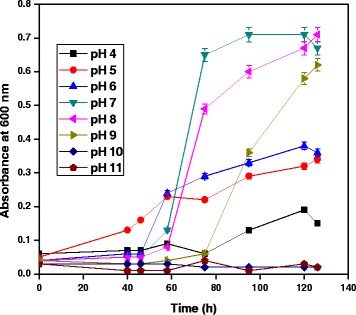
**Effect of pH for growth of** ***A. baumannii*** **using diesel oil at 35°C.**

Effect of pH for growth of *A. baumannii* with diesel oil was evaluated. The *A. baumannii* showed maximum growth in pH 7. The growth of the bacterial species was decreased while decreasing or increasing the pH. Hence it is understood that neutral pH is required for optimum growth of bacteria, acidic and basic conditions did not favour the growth of *A. baumannii*. Figure 1 shows the growth of bacterial species at different pH in MSM containing 1% diesel oil. According to Whang et al. [10] microbial growth and diesel biodegradation was found to be at a pH 7.2, while decreasing or increasing the pH reduced the degradation efficiency considerably. Xia et al. [11] studied the effect of pH on diesel degradation through the response surface methodology (RSM) using a central composite design and they found the and the optimal biodegradation conditions of diesel oil was pH 7.4. According to Luo et al. [12] at pH level of 7 P*seudomonas* sp. strain F4 showed efficient diesel oil degradation potential. Hence, the optimization of pH is very important for the enhanced growth of bacteria and also for selection of effective bioremediation strategy. Sathishkumar et al. [13] reported that the optimum pH for the degradation of crude oil by individual bacterial strains and a mixed bacterial consortium was found to be 7.

### Effect of temperature

**Figure 2:**
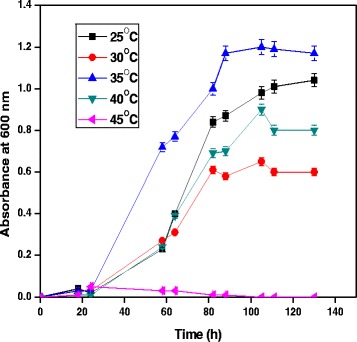
**Effect of temperature for growth of** ***A. baumannii*** **using diesel oil at pH 7.**

Effect of temperature for growth of *A. baumannii* with diesel oil was evaluated. We found that temperature is an important factor that affects the diesel degradation potential by bacteria. Mnif et al. [14] found that 30°C was the optimum condition for the degradation of diesel by *Bacillus subtilis* SPB1. Same time, the diesel oil-degrading ability of *Pseudomonas* sp. strain F4 was reported to be 37°C [12]. The present study analyzed the optimum temperature for the degradation of diesel oil and it was found maximum at 35°C. The minimum growth was observed at 45°C. Maintenance of temperature is as important as pH which strongly affects bacterial growth. The growth of *A. baumannii* was directly proportional to diesel oil degradation, since the medium with diesel oil as the sole source of carbon. The degradation efficiency decreased greatly with the increase of temperature. Figure 2 shows the growth of bacterial species at different temperature in MSM containing 1% diesel oil.

### Effect of initial hydrocarbon concentration

**Figure 3:**
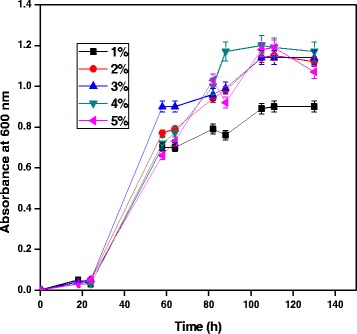
**Effect of initial hydrocarbon concentration for growth of** ***A. baumannii*** **using diesel oil at pH 7 and 35°C.**

Effect of initial hydrocarbon concentration for growth of *A. baumannii* with diesel oil was evaluated. Initial hydrocarbon concentrations of 1-5% were used for this degradation studies in which MSM containing 4% of diesel oil showed maximum growth of *A. baumannii*, as shown in Figure 3. Minimum growth of bacterial species was observed in 1% concentration of initial hydrocarbon. At high diesel oil concentration, diesel oil provided a better carbon source for the growth of bacteria Luo et al. [15]. The growth of the bacterial species was increased with increase in diesel concentration. The bacterial species did not possess higher growth at above 4% diesel oil concentration. The reason for decreased consumption of diesel oil at high concentration may be attributed due to stress of hydrocarbons on bacterial species.

### Effect of initial inoculum size

**Figure 4:**
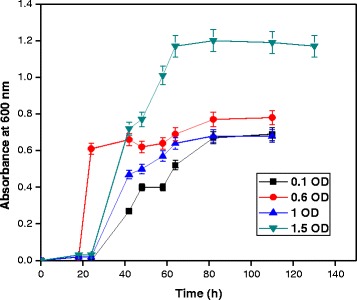
**Effect of initial inoculum concentration for growth of** ***A. baumannii*** **using diesel oil at pH 7 and 35°C.**

Effect of initial inoculum size for growth of *A. baumannii* with diesel oil was evaluated. MSM medium was inoculated with initial inoculum concentration of 0.1, 0.6, 1.0 and 1.5 optical density measured at 600 nm. Figure 4 shows the effect of inoculum quantity of bacterial species on diesel oil degradation. The removal of hydrocarbon mainly depends on the capabilities of the microorganisms [16]. When inoculum of *A. baumannii* increased up to 1.5 optical density, lag period for growth of strain decreased and subsequently resulted in higher growth indicates higher diesel oil degradation as depicted in Figure 4. *Pseudomonas* sp. showed efficient diesel degradation efficiency when the inoculums size was adjusted to 2%(v/v) [12]. Luo et al. [15] shows that when the bacteria concentration was 4 × 10^7^ cells/mL, the cell density and biodegradation rate were all the highest after seven days incubation. The removal of hydrocarbon mainly depends on the capabilities of the microorganisms. Lakshmi et al. [17] reported that immobilized (18.6 × 10^6^ cells/mL) and free cells of *Mycoplana* sp. MVMB2, a soil isolate was able to degrade 95% phenanthrene within 72 h and 120 h respectively.

### Effect of NaCl concentrations

**Figure 5:**
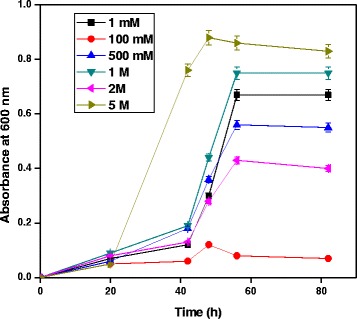
**Role of NaCl concentration for growth of** ***A. baumannii*** **using diesel oil at pH 7 and 35°C.**

Effect of NaCl concentration for growth of *A. baumannii* with diesel oil was evaluated. Metabolic capacities of the bacterial species with respect to salinity were tested for degradation of diesel oil. *A. baumannii* was degraded in the presence of 1 mM, 100 mM, 500 mM, 1 M, 2 M and 5 M NaCl concentration. Increase in NaCl concentration increases the growth of the bacteria in MSM containing 4% diesel oil. Figure 5 shows that variations in salinity had a strong influence on biodegradation which progressively increased when salinity increased. This would be particularly appropriate for removal of stranded oil on beaches or intertidal areas where bacterial species would have to survive exposure to high concentrations of sodium chloride, or in estuarine areas where salinity gradients occur. Mnif et al. [18] isolated a novel aromatic-degrading bacterium from a geothermal oil field under saline and thermophilic conditions and the optimum NaCl concentration for degradation was found to be 10 g/L.

### Role of carbon and nitrogen sources

**Figure 6:**
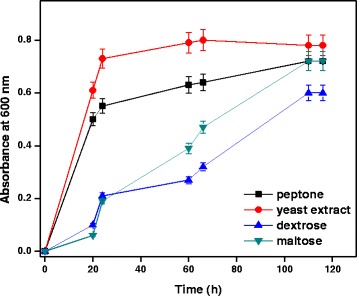
**Effect of carbon and nitrogen sources for growth of** ***A. baumannii*** **using diesel oil at pH 7 and 35°C.**

Role of carbon and nitrogen sources for growth of *A. baumannii* with diesel oil was evaluated. Various carbon and nitrogen sources such as dextrose, maltose, yeast extract and peptone (0.1%) were added to the MSM medium containing 4% diesel oil as nutrient addition for diesel oil degradation, amongst them peptone and yeast extract were found to enhance diesel oil degradation). Prathima Devi et al [19] reported that addition of external nutrients enhance the degradation of crude petroleum sludge. As shown in Figure 6, growth of *A. baumannii* was increased in the presence of peptone within 120 h. Consequently, peptone was selected as the nitrogen source for further studies. The addition of glucose caused a significant difference in the ability of the diesel degraders to break down diesel of up to 84% [20]. Zahed at al. [21] reported that crude oil removal of 69.5% was observed in presence of 16.05 mg/L nitrogen. Liu et al. [22] reported that addition of carbon source increased the rate of degradation of hydrocarbon by *Rhodococcus* sp. demonstrated that minerals or supplementary carbon substrates increase the rate of biodegradation. NH_3_ may be used by microbes as a source of N which undergo nitrification and volatilize. NO_3_^−^+NO_2_^−^ may be used by microbes as a source of N, undergo denitrification [23]. Carbon sources were not utilized by the bacterial species at earlier stage and there was minimum growth in the presence of carbon sources.

### Effect of chemical surfactants

**Figure 7:**
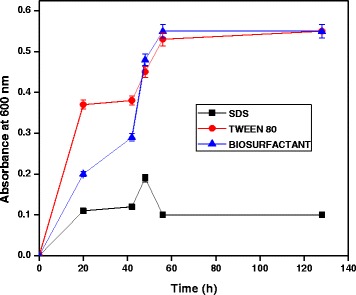
**Role of surfactants for growth of** ***A. baumannii*** **using diesel oil at pH 7 and 35°C.**

The effect of surfactants (chemical and biological) on diesel oil degradation by bacterial strain is depicted in Figure 7. Tween 80, SDS and biosurfactant enhanced the growth of bacterial species within 72 h compared to the growth of bacterial species without surfactants. However, addition of non-ionic surfactants did not show any significant increase in diesel oil degradation in comparison to control (without surfactants). Bautista et al. [24] had also reported that Tween 80 was the best amongst non-ionic surfactants in improving the degradation of PAHs.

Possibly, *A. baumannii* produced biosurfactants, which increased pseudo solubilization of diesel oil in the medium.

### GC-MS analysis

**Figure 8:**
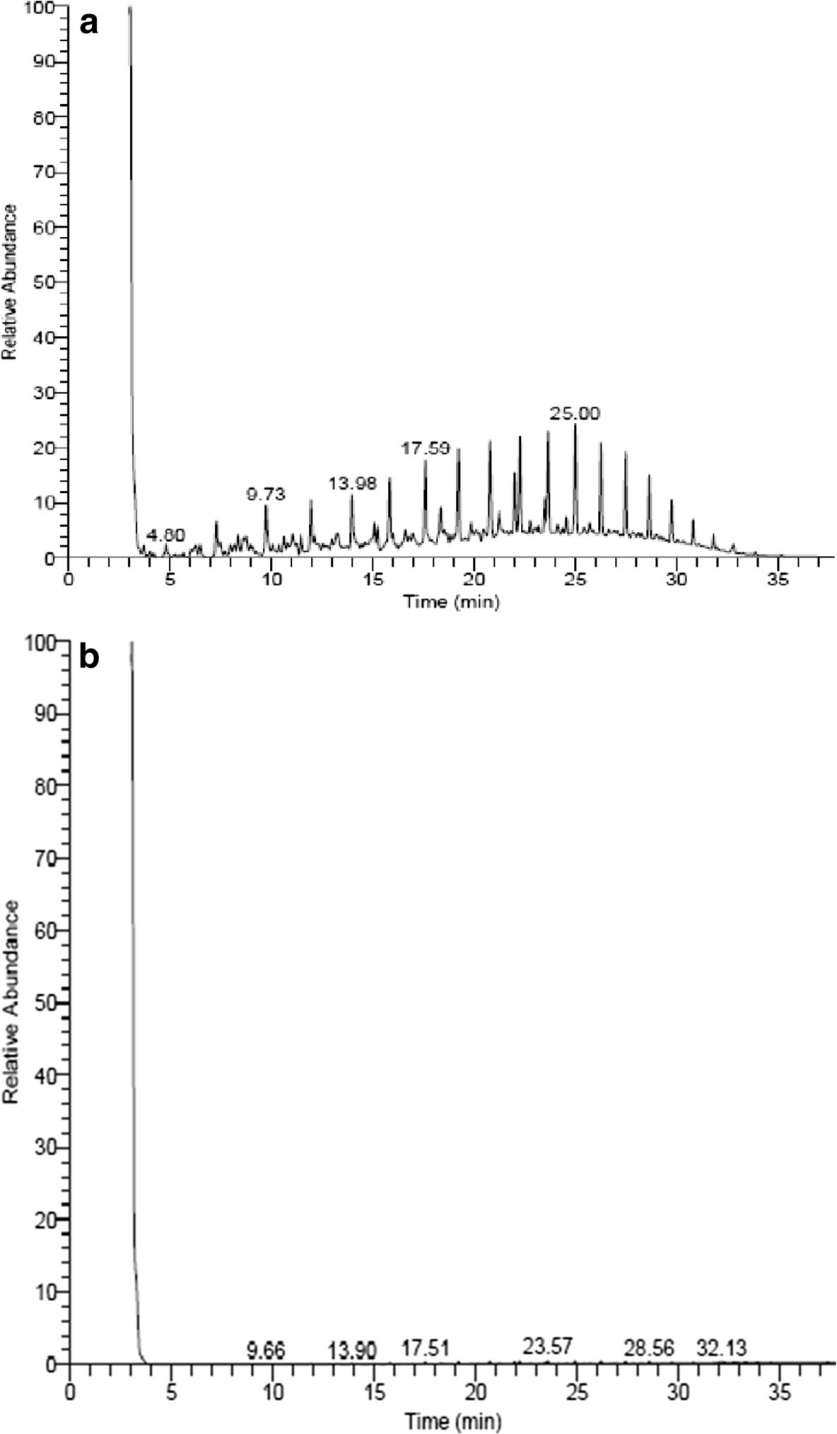
**a: GC-MS profile for control diesel oil and b: GC-MS profile for diesel oil after biodegradation by** ***A. baumannii*** **.**

The biodegradation of diesel oil by *A. baumannii* was confirmed by GC-MS analysis. GC-MS chromatogram showed reduction in the intensity of diesel oil peaks after the degradation with *A. baumannii* when compared with control diesel oil (Figure 8). The study shows that *A. baumannii* was able to degrade >99% of the diesel oil within 5 days of incubation at initial pH 7 and 37°C. The previous study achieved degradation of 95.01% diesel by *Trichosporon asahii* [25]. Similarly Chandran and Das [26] reported that free cells of *C. tropicalis* able to degrade 80% of the diesel oil over a period of one week.

## Conclusion

The present study focused on the degradation of diesel oil by a bacterial species isolated from diesel contaminated site. The success of oil bioremediation depends on our ability to optimize various physical, chemical, and biological conditions in the contaminated environment. Here, *A. baumannii* was able to degrade more than 99% of diesel oil at pH 7, 35°C and initial hydrocarbon concentration of 4%. The present study may be applied for the efficient removal of diesel oil containing industrial effluents released from petroleum refineries.
